# Regulation of Satiety Quiescence by Neuropeptide Signaling in *Caenorhabditis elegans*

**DOI:** 10.3389/fnins.2021.678590

**Published:** 2021-07-15

**Authors:** Mei Makino, Enkhjin Ulzii, Riku Shirasaki, Jeongho Kim, Young-Jai You

**Affiliations:** ^1^Neuroscience Institute, Department of Biology, Nagoya University, Furo-cho, Japan; ^2^Department of Biological Sciences, Inha University, Incheon, South Korea; ^3^Center for Hypothalamic Research, University of Texas Southwestern Medical Center, Dallas, TX, United States

**Keywords:** satiety, metabolism, quiescence, TGFβ, neural circuit, cyclic GMP

## Abstract

Sleep and metabolism are interconnected homeostatic states; the sleep cycle can be entrained by the feeding cycle, and perturbation of the sleep often results in dysregulation in metabolism. However, the neuro-molecular mechanism by which metabolism regulates sleep is not fully understood. We investigated how metabolism and feeding regulate sleep using satiety quiescence behavior as a readout in *Caenorhabditis elegans*, which shares certain key aspects of postprandial sleep in mammals. From an RNA interference-based screen of two neuropeptide families, RFamide-related peptides (FLPs) and insulin-like peptides (INSs), we identified *flp-11*, known to regulate other types of sleep-like behaviors in *C. elegans*, as a gene that plays the most significant role in satiety quiescence. A mutation in *flp-11* significantly reduces quiescence, whereas over-expression of the gene enhances it. A genetic analysis shows that FLP-11 acts upstream of the cGMP signaling but downstream of the TGFβ pathway, suggesting that TGFβ released from a pair of head sensory neurons (ASI) activates FLP-11 in an interneuron (RIS). Then, cGMP signaling acting in downstream of RIS neurons induces satiety quiescence. Among the 28 INSs genes screened, *ins-1*, known to play a significant role in starvation-associated behavior working in AIA is inhibitory to satiety quiescence. Our study suggests that specific combinations of neuropeptides are released, and their signals are integrated in order for an animal to gauge its metabolic state and to control satiety quiescence, a feeding-induced sleep-like state in *C. elegans*.

## Introduction

From simple invertebrates to complicated humans, most animals exhibit a behavioral state of sleep. Circadian rhythm can be entrained by feeding, and perturbation of the rhythm often results in obesity. In addition, the neuropeptide orexin regulates both sleep and feeding, suggesting that the two behaviors are linked (Bass and Takahashi, 2010; Marcheva et al., 2010). However, the role of metabolism in sleep and the underlying neuronal mechanisms that connect metabolism to sleep are not fully understood.

*Caenorhabditis elegans*, with its powerful genetics and simple nervous system, emerges as an ideal model to study neuro-molecular mechanisms underlying sleep. Two types of sleep, developmentally timed sleep (DTS), and stress-induced sleep (SIS), share fundamental aspects of sleep in other animals (Flavell et al., 2020). Additionally, we reported that the *C. elegans* behavioral state satiety quiescence mimics certain aspects of post-prandial sleep in mammals (You et al., 2008). After feeding, rodents exhibit behavioral sequence of satiety: termination of meals, reduction of locomotion, and sleep (Gibbs et al., 1973; Antin et al., 1975). *C. elegans* also becomes quiescent after feeding. This quiescence induced by satiety is dependent on the animal’s metabolic state. If the animal is undernourished, either by being deprived of food, or by having defects in the feeding or digestion processes, the animal shows little quiescence. During satiety quiescence, an animal is inactive and exhibits a sleep-like posture. The longer an animal is starved, the more quiescent it becomes after being refed. The animal’s response to touch is reduced during satiety quiescence; the animal no longer exhibits a full escape response but instead, returns to quiescence almost immediately after being touched, suggesting that their touch perception is dampened during satiety quiescence. These observations suggest satiety quiescence contains several key components of behavioral state of sleep and thus provides us a unique readout to study how metabolism might regulate sleep or sleep-like states.

Satiety quiescence is regulated by insulin, cGMP, and TGFβ signaling. In *C. elegans*, the three signaling pathways regulate dauer formation during development, which is also critically dependent on the animal’s metabolic state. Lack of any of these three signals drives the animal to enter the dauer state, a dormant stage to survive unfavorable environment (Hu, 2007). The wrong decision of whether to enter the reproductive cycle or the dauer state is fatal. The animal either would not survive if it enters to reproductive cycle in a harsh environment or would be outcompeted while the other animals prosper if it becomes dauer in a nutritious environment. The fact that the same three pathways control both the critical developmental choice and satiety quiescence, indicates that these three pathways are used to ensure the animals of metabolic wellbeing.

We found that TGFβ released from the head sensory neuron pair ASI is necessary for satiety quiescence. Lack of TGFβ signal reduces quiescence and increases fat storage, again linking metabolism and sleep by a single molecular pathway (Gallagher et al., 2013b). The conserved roles of cGMP and TGFβ signaling in feeding have also been discovered in mammals (Valentino et al., 2011; Tsai et al., 2013, 2018; Folgueira et al., 2018), suggesting a similar set of molecules regulate metabolism and sleep in many animals. Satiety quiescence requires intact fat metabolism mediated by the SREBP-SCD (sterol regulatory element-binding protein – stearoyl-CoA desaturase) pathway, indicating that a communication between the nervous system and the organs of energy storage is necessary (Hyun et al., 2016).

Neuropeptides (NP) are the major signaling molecules that control feeding and energy homeostasis in most of the animals including invertebrates (Li and Kim, 2008; Nassel et al., 2019). *C. elegans* has a total of 121 neuropeptides divided into three families: 34 RFamide-related peptides (FLP) characterized by a C-terminal Arg-Phe-amide motif, 47 neuropeptide-like proteins (NLP), and 40 insulin-like proteins (INS) (Li and Kim, 2008). We and others have demonstrated that many of these peptides serve conserved functions in metabolism and feeding. NLP-24, a *C. elegans* opioid capable of binding to human μ-opioid receptors, regulates fasting responses (Cheong et al., 2015). FLP-7, a *C. elegans* tachykinin, centrally regulates peripheral fat storage by controlling the transcription level of a hormone-sensing lipase in periphery (Palamiuc et al., 2017). NLP-75 the *C. elegans* oxytocin/vasopressin regulates reproductive behavior and gustatory associative learning (Beets et al., 2012; Garrison et al., 2012). NLP-38 is also critical for the animal to form a memory to avoid salt concentration associated with starvation condition (Peymen et al., 2019).

RF-amide peptides play roles in diverse behaviors such as social interaction, reproduction, and feeding (Parhar et al., 2016; Quillet et al., 2016). In zebrafish, the RFamide neuropeptide VF (NPVF) and the *npvf*-expressing neurons are necessary and sufficient to promote sleep (Lee et al., 2017). In *C. elegans*, both FLPs and NLPs regulate sleep: FLP-18, NLP-2, NLP-22, and NLP-14 regulate DTS and FLP-13, FLP-24, and NLP-8 do SIS (Nelson et al., 2013; Nagy et al., 2014; Nath et al., 2016; Iannacone et al., 2017; Honer et al., 2020; Van der Auwera et al., 2020).

*Caenorhabditis elegans* has 40 insulin-like ligands all of which act on the insulin receptor DAF-2 (Murphy and Hu, 2013). The pathway has conserved components, including positive regulators of PI3K (AGE) and AKT (AKT) as well as the negative regulators of PTEN (DAF-18) and FOXO (DAF-16). The insulin pathway regulates vast arrays of physiological processes, including L1 arrest, dauer decision, fat and sugar metabolism and longevity (Murphy and Hu, 2013). The 40 *ins* genes redundantly function as either agonists or antagonists to the DAF-2 receptor and can regulate a specific process such as L1 arrest or fat metabolism or all of the processes (Zheng et al., 2018). Among the 40 *ins* genes, *ins*-*1* is most similar to the human insulin in its sequence and antagonizes DAF-2 insulin receptor (Pierce et al., 2001). INS-1 plays roles in salt conditioning, olfactory feedback, and thermotaxis plasticity by starvation (Tomioka et al., 2006; Chalasani et al., 2010; Takeishi et al., 2020). These studies indicate that INS peptides play critical roles in conveying the animal’s metabolic states. Furthermore DAF-16, the *C. elegans* FOXO ortholog and the major downstream target of insulin signaling, is required for sleep homeostasis and is essential for DTS (Driver et al., 2013; Bennett et al., 2018). However, despite its conserved role in feeding and metabolism, the roles of insulin signaling in sleep has not been systemically tested in *C*. *elegans*.

Based on the conserved roles of neuropeptides in regulation of homeostasis, such as in energy balance and in sleep, we performed an RNA interference-based screen of two neuropeptide families, FLPs and INSs. Among the 28 tested *flp* genes, RNA interference (RNAi) of 10 FLPs altered satiety quiescence, while *flp-11*, known to regulate other types of sleep-like behaviors in *C. elegans* plays the most important role. *flp-11* mutants show reduction in satiety quiescence, whereas over-expression lines show enhancement. FLP-11 acts in the interneuron RIS; a mutant that carries a mutation in the gene *aptf-1*, a transcription factor that functions in RIS, is also defective in satiety quiescence. From a genetic analysis, we found that FLP-11 acts potentially upstream of the cGMP signaling and downstream of the TGFβ pathway, suggesting a potential neural circuit. Among the 28 tested *ins* genes, *ins-1*, known to play a significant role in starvation-associated behavior working in AIA interneurons and receiving input from ASI neurons (Tomioka et al., 2006), is inhibitory to satiety quiescence. Our results could suggest a neural circuit where internal nutrient status is integrated to generate an appropriate behavioral output such as satiety quiescence.

## Materials and Methods

### Strains

The wild-type strain was *C. elegans* variant Bristol, strain N2. Mutant strains were, GR1396 *eri-1 IV; lin-15b X*, VC2324 *flp-6(ok3056) V*, RB1990 *flp-7(ok2625) X*, PT501 *flp-8(pk360) X*, RB2067 *flp-9(ok2730) IV*, RB1989 *flp-10(ok2624) IV*, VC1669 *aptf-1(gk794) II*, HBR507 *flp-11(tm2706) X*, RB1863 *flp-12(ok2409) X*. These strains are available from the *Caenorhabditis* Genetics Center (CGC). YJ233 *flp-6(ok3056) V*, YJ234 *flp-7(ok2625) X*, YJ235 *flp-9(ok2730) IV* and YJ236 *flp-10(ok2624) IV* were outcrossed 2–4 times from the original strain. The *flp-11* overexpression (OE) lines, YJ258 and YJ259, were generated by injecting the reporter *Psur-5::mCherry* with the target plasmid (pPD95.77, *Pflp-11::flp-11::GFP*) carrying the *flp-11* gene into N2 strain animals (Sunny Biotech). The double mutant YJ262 *flp-11(tm2706) X*; *egl-4(ks62) IV* was generated by crossing HBR507 *flp-11(tm2706) X* with FK234 *egl-4(ks62) IV*. YJ 263 *flp-11(tm2706)* X; *egl-4(ad450sd) IV* was generated by crossing HBR507 *flp-11(tm2706) X* with DA521 *egl-4(ad450sd) IV*. The double mutant of *flp-11* OE; *daf-7* was generated by crossing YJ258 with CB1372 *daf-7(e1372) III*. All animals were maintained at 20°C on *Escherichia coli* strain HB101 unless indicated otherwise.

### RNAi Screening

**FIGURE 1:**
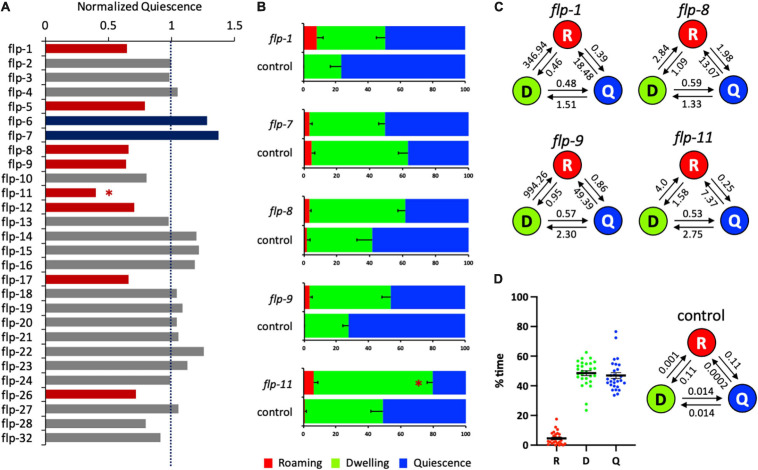
Several *flp* genes regulate satiety quiescence. **(A)** The normalized time in quiescence after knockdown of individual *flp* genes by RNA interference. The percent time in quiescence of each *flp* is normalized to the percent time in quiescence of the concurrent control (RNAi vector only). Each RNAi was performed with the sample number of 15–18 of tested animals with 7–9 control animals. Red bars indicate that RNAi of the gene reduced satiety quiescence, whereas blue bars indicate that RNAi of the gene enhanced satiety quiescence with an approximate cut-off by ±20% of the normalized value (see Table 1). **(B)** The individual data of the *flp* genes including whose RNAi showed significant changes in satiety quiescence. After two generations of feeding RNAi, the age-synchronized 1-day-old adults were tested for their locomotive activity by measuring the percent time they spent in roaming, dwelling and quiescence. **(C)** The transition rates of the animals treated with RNAi of *flp-1, flp-8, flp-9*, and *flp-11*. The circles of R, D, and Q represent each state of roaming, dwelling, and quiescence, respectively. The rates are shown above the arrow that indicates the direction of transition. **(D)** Distribution of the values of each state (left) and average transition rates (right) of the 28 independently tested RNAi control groups. (^∗^*p* < 0.009 by Student’s *t*-test after the Bonferroni correction. The error bars indicate SEM).

Among 34 *flp* genes and 40 *ins* genes, 28 available clones of *flp* genes and *ins* genes from Ahringer feeding library were tested by bacteria-mediated feeding RNAi (Fraser et al., 2000). The plates containing NGM agar with 1 mM IPTG and 50 μg/ml carbenicillin were inoculated with bacterial cultures grown 16–18 h for each target gene. HT115 bacteria, an RNase III-deficient *E. coli* was used. The strain GR1396 (*eri-1 IV; lin-15B X*) was used to enhance RNAi sensitivity (Ruvkun G., personal communication). Three L4 stage animals were transferred to the plates and 36 h later the adults were removed. Another 36 h later, the progeny L4 animals were picked to perform satiety quiescence assay. For each test, 7–9 concurrent control animals treated with empty vector (L4440) containing RNAi bacteria and 15–18 animals treated with an RNAi containing bacteria were used. Except for the RNAi clones whose treatment resulted in no significant difference compared to the control (gray bars in Figures 1, 3), the experiment was repeated at least twice.

**TABLE 1:** Normalized values of each state of roaming, dwelling and quiescence, the sample size, and statistical significance of 28 *flp* genes.

**Genes**	**Normalized values**			***p-*value (by Student’s *t*-test)**			**Sample size (*N*)**
	**R**	**D**	**Q**	**R**	**D**	**Q**	**Control, RNAi**
*flp-1*	1244.13	1.81	0.65	0.2522	0.0469	0.0129	6,16
*flp-2*	0.48	1.02	0.99	0.3047	0.9007	0.9693	9, 17
*flp-3*	12,250.78	1.01	0.98	0.4889	0.9443	0.9356	8, 16
*flp-4*	0.32	0.95	1.05	0.3828	0.8373	0.8190	7, 12
*flp-5*	2.32	1.24	0.79	0.5287	0.2114	0.2197	9, 14
*flp-6*	0.05	0.80	1.28	0.0053	0.1341	0.1117	9, 17
*flp-7*	0.72	0.79	1.37	0.6420	0.0549	0.0506	9, 18
*flp-8*	1.78	1.46	0.66	0.5139	0.0285	0.0381	8, 17
*flp-9*	3858.48	1.82	0.64	0.2391	0.0117	0.0123	6, 17
*flp-10*	2.96	1.16	0.80	0.2325	0.3999	0.2217	9, 16
*flp-11*	6.75	1.53	0.40	0.1943	0.0013	0.0003	9, 16
*flp-12*	2.87	1.00	0.71	0.0964	0.9771	0.1906	8, 16
*flp-13*	0.64	1.06	0.98	0.6067	0.6132	0.9115	9, 17
*flp-14*	0.32	0.90	1.19	0.1914	0.5484	0.3776	8, 16
*flp-15*	0.55	0.91	1.22	0.4113	0.5147	0.2811	9, 18
*flp-16*	0.55	0.90	1.18	0.3728	0.6383	0.4997	8, 17
*flp-17*	1.57	1.16	0.66	0.4339	0.4270	0.2113	8, 18
*flp-18*	1.53	0.94	1.04	0.6093	0.6603	0.8869	9, 17
*flp-19*	1.35	0.89	1.09	0.7112	0.5022	0.6450	8, 17
*flp-20*	2.65	0.90	1.04	0.4253	0.3581	0.8498	9, 17
*flp-21*	1.42	0.93	1.05	0.7293	0.7419	0.7803	8, 16
*flp-22*	1.92	0.81	1.26	0.5146	0.1625	0.3602	9, 17
*flp-23*	1.94	0.78	1.12	0.3716	0.1149	0.6500	9, 16
*flp-24*	0.34	1.29	0.99	0.1783	0.2635	0.9689	7, 17
*flp-26*	1.05	1.23	0.72	0.9566	0.1652	0.1675	8, 15
*flp-27*	0.52	1.01	1.06	0.2975	0.9746	0.7852	8, 15
*flp-28*	1.18	1.09	0.79	0.7382	0.4795	0.4280	9, 18
*flp-32*	1.96	0.97	0.91	0.3977	0.8702	0.7335	9, 18

**FIGURE 2:**
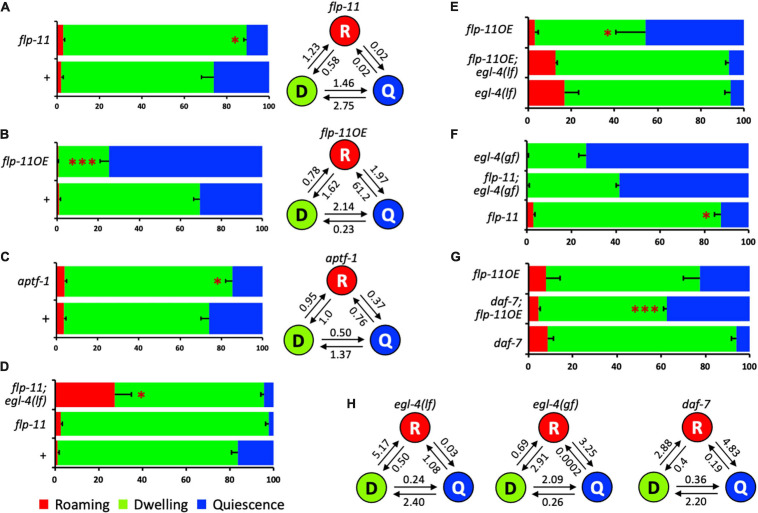
The potential mechanisms of *flp-11* in satiety quiescence. **(A)** *flp-11* mutants show reduced satiety quiescence, confirming the RNAi result. The percent time and the normalized transition rates among the three states are shown. **(B)** An overexpression line of *flp-11* shows enhanced satiety quiescence. The data is a representative of two overexpression lines. The percent time and the normalized transition rates among the three states are shown. **(C)** *aptf-1*, a transcription factor acting in RIS that promotes other types of sleep behavior in *C. elegans*, is also required for satiety quiescence. The percent time and the normalized transition rates among the three states are shown. **(D)** Both *flp-11* and *flp-11*; *egl-4* loss of function mutants are defective in satiety quiescence [for comparison to *egl-4* single mutant, see **(E)**]. Considering the roaming behavior of the *flp-11*; *egl-4* mutants shown in the red bar, *egl-4* could be epistatic to *flp-11*. **(E)** Overexpression of *flp-11* does not suppress the defect in the *egl-4* loss of function mutants. **(F)** A gain-of-function allele of *egl-4*(gf) rescued *flp-11* loss of function, suggesting that *egl-4* could act downstream of *flp-11*. **(G)** *flp-11* overexpression suppresses the defect in quiescence of *daf-7* mutant. **(H)** The transition rates of *egl-4(if)*, *egl-4(gf)*, and *daf-7* mutants. (^∗^*p* < 0.05, ^∗∗∗^*p* < 0.001 by Student’s *t*-test. The error bars indicate SEM).

**FIGURE 3:**
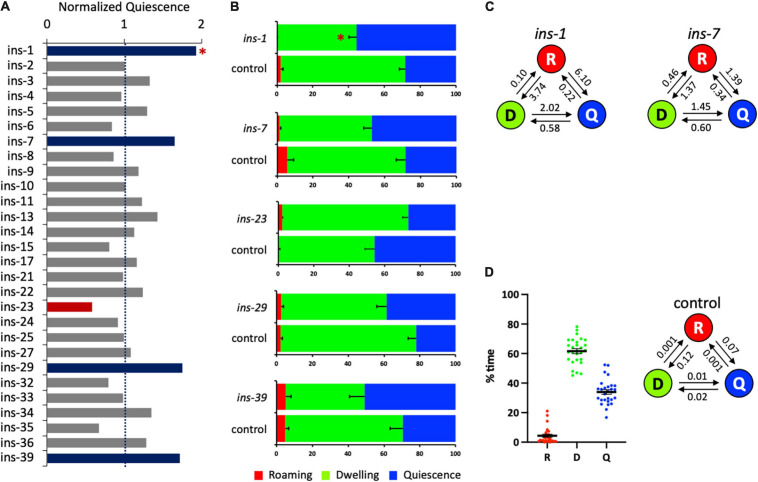
Several *ins* genes regulate satiety quiescence. **(A)** The normalized time in quiescence after knockdown of individual *ins* gene by RNA interference. The percent time in quiescence of each *ins* is normalized to the percent time in quiescence of the concurrent control (RNAi vector only). Each RNAi was performed with the sample number of 15–18 of tested animals with 7–9 control animals. The experiment was repeated at least three times. Red bars indicate that RNAi of the gene reduced satiety quiescence, whereas blue bars indicate that RNAi of the gene enhanced satiety quiescence. **(B)** The individual data of the *ins* genes whose RNAi resulted in changes in satiety quiescence. After two generations of feeding RNAi, the age-synchronized 1-day-old adults were tested for their locomotive activity by measuring the percent time they spent in roaming, dwelling, and quiescence. **(C)** The transition rates of the animals treated with RNAi of *ins-1* and *ins-7*. **(D)** Distribution of the values of each state (left) and average transition rates (right) of the 28 independently tested RNAi control groups. (^∗^*p* < 0.009 by Student’s *t*-test after the Bonferroni correction. The error bars indicate SEM).

### Satiety Quiescence Assay

Satiety quiescence was measured with an automated method using a nine-video camera monitoring system, as previously described (Gallagher et al., 2013a). For the RNAi screening, on day 1, approximately 20 L4s of the F1 generation were picked and placed on the new RNAi plates with 100 μl of the RNAi bacteria and grown for 24 h. The next day, the RNAi bacteria culture was centrifuged at 4000 RPM for 3 min and the supernatant was removed. The pellet was mixed three 1:1 dilutions of bacteria with M9 buffer. After that, 5 μl of the diluted bacteria was added to each of a 35 mm plate and dried completely, and then a single animal was transferred to each plate. One plate was placed under each video camera of the nine-camera system we built (Gallagher et al., 2013a). Measuring satiety quiescence of mutant strains was performed the same way, except instead GR1396 strain, the outcrossed mutant line was tested while N2 was used as control, and instead of the RNAi bacteria, *E. coli* HB101 containing mCherry was used for food. Once all nine animals were in focus, the LED light was turned off for 30 min to give the animals time to recover from the transfer. After the 30 min, the LED light was turned on and the image was captured at the rate of 1 frame/second for 30 min using the Point Grey’s FlyCap2 software. The centroid position of each worm was identified in each image using custom written software and the change in centroid position between frames was used to calculate worm movement. The movement data was analyzed using a custom written Hidden Markov Model based program (Gallagher et al., 2013a) and the percent time for each of three behavioral states, roaming, dwelling and quiescence, and the transition rates were calculated.

### Statistics

The Bonferroni correction (0.05/56 = 0.0009) was applied to correct for multiple comparisons for two independent comparisons for each RNAi experiment (28 × 2 for two states) and assigned the significance accordingly.

## Results

### An RNAi Screen of 28 *flp* Genes Identified FLP-11 in RIS as a Major Regulator of Satiety Quiescence

To investigate the roles of *flp* genes in satiety quiescence, we performed feeding RNAi and individually knocked down 28 genes whose clones were available to us from the Ahringer library (Fraser et al., 2000). Satiety quiescence is detected under two conditions: non-fasted but fed with high quality food and fasted then refed. The animals show consistent quiescence in both settings, although it is higher in fasted and refed animals compared to non-fasted (You et al., 2008). The non-fasted condition allowed us to rapidly perform the RNAi screen for satiety quiescence in a reliable manner (Hyun et al., 2016). The animals were fed with the RNAi clone of each neuropeptide gene from L4s (the 1^st^ generation). The L4s of the 2^nd^ generation were picked and tested next day as young adults. Throughout the assay, the animals were continuously fed with the RNAi bacteria (see section “Materials and Methods”).

When satiety quiescence was measured using an automated system (Gallagher et al., 2013a), the knockdown of 10 *flp* genes showed altered satiety quiescence (Figure 1A and Table 1). Previous studies defined several behavioral states based on locomotive activities (Fujiwara et al., 2002; Gray et al., 2005; Ben Arous et al., 2009; Gallagher et al., 2013a; Flavell et al., 2020). Roaming is a state when an animal moves straight in a relatively high speed to explore, dwelling is a state when an animal moves back and forth in a low speed to exploit, and quiescence is a state when an animal is not moving. When we examined individual locomotive activity regarding the percent time the animal spent in each of three behavioral states, RNAi of five *flp* genes (*flp-1, 7, 8, 9*, and *11*) reduced the percent time in quiescence, although the only one that reached statistical significance after the correction was *flp-11* (Figure 1B). RNAi of *flp-7*, a *C. elegans* tachykinin implicated in nutrient sensing and lipid metabolism (Palamiuc et al., 2017), shows a tendency to enhance satiety quiescence. We also examined the normalized transition rates between each state. Transition rates are analogous to rate constants of first-order chemical reactions, and in this case reflect the tendency of the animal to switch from one state to another (Gallagher et al., 2013a). Due to the very low percent time that animals spend in roaming, there are extremely small values of transition rates entering roaming (for example, Figure 1D, 0.001 for dwelling to roaming, 0.0002 for quiescence to roaming), and so the normalized transition rates between roaming and the other two states are often not meaningful. However, the transition rates between dwelling and quiescence could imply the duration of each quiescence bout because more frequent switching could result in a short duration. When normalized to the transition rates of concurrent controls, RNAi of the five *flps* reduced the transition rates from dwelling to quiescence but increased the rates from quiescence to dwelling, suggesting their quiescence bouts are likely reduced (Figure 1C).

Satiety quiescence is extremely variable especially when the animals are tested under the non-fasted condition. Therefore, we always run concurrent controls which were picked as L4s among the progeny from the same mother, cultivated with the control RNAi bacteria under the exact same condition as the group treated with the testing RNAi. When we perform the experiment, we provide as consistent conditions as possible for the temperature, humidity, and the time of the day to ensure that the errors of the control are minimum. To assess the variation, we combined the data from all 28 control groups from each independent experiment (where each control group consists of 6–9 animals) and analyzed the distribution of the percent time of roaming (R), dwelling (D), and quiescence (Q) and found most of the values are within 20% of variation with a few outliers (Figure 1D). We also calculated the average values of the transition rates of the 28 control groups (Figure 1D). The data show that the animals in the control groups have an equal tendency to switch from dwelling to quiescence and from quiescence to dwelling. The animals spent most of their time dwelling or quiescent as the transition rates of either dwelling or quiescence to enter roaming are extremely low.

Among the five, *flp-11* RNAi reduced satiety quiescence most. *flp-11* is known to regulate two types of sleep, DTS and SIS, in *C. elegans*. When we tested *flp-11* mutants, they also showed reduction in satiety quiescence, confirming the RNAi result (Figure 2A). In contrast to the *flp-11* loss-of-function mutant, a *flp-11* overexpression (*flp-11OE*) under its own promoter enhanced satiety quiescence (Figure 2B). *flp-11* mutants increase transition rates from quiescence to dwelling, whereas *flp-11OE* decreases it, suggesting *flp-11* is required for long bouts of satiety quiescence. *flp-11* regulates DTS and SIS through its action in an interneuron RIS. To examine whether satiety quiescence is also regulated by the *flp-11* action in RIS, we tested a mutant of *aptf-1*, which is necessary for RIS function (Turek et al., 2016). The mutant showed reduced quiescence, as the *flp-11* mutant did, suggesting that *flp-11* regulates satiety quiescence through its action in RIS (Figure 2C).

### ELG-4 Likely Acts Downstream of FLP-11 to Regulate Satiety Quiescence

Satiety quiescence requires function of *egl-4*, a cGMP-dependent protein kinase (You et al., 2008). To investigate whether *flp-11* genetically interacts with *egl-4*, we generated three different double mutants: (1) *flp-11* loss-of-function with *egl-4* loss-of-function, (2) *flp-11OE* with *egl-4* loss-of-function, and (3) *flp-11* loss-of-function with *egl-4* gain-of-function. The double mutant of *flp-11* loss-of-function with *egl-4* loss-of-function shows a similar phenotype to that of *egl-4* loss-of-function single mutants – reduced percent of quiescence and enhanced of roaming (Figures 2D,E and Table 2). In addition, overexpression of FLP-11 does not rescue the loss-of-function mutant of *egl-4*. Finally, *egl-4* gain-of-function restored satiety quiescence to *flp-11* mutants (Figure 2F). Taken together, these results suggest that EGL-4 can act downstream of FLP-11. When we examined the normalized transition rates of *egl-4* loss-of-function, gain-of-function and *daf-7* mutants, they also showed the changes in the transition rates between dwelling and quiescence, consistent with their changes in percent time the animals spent in the two states (Figure 2H).

**TABLE 2:** Normalized values of each state of roaming, dwelling and quiescence, the sample size, and statistical significance of *flp-11*, *flp-11OE*, *egl-4(lf)*, *egl-4(gf)*, *daf-7*, and the double mutants.

**Comparison (1, 2)**	**Normalized values**			***p*-value**			***N* (1, 2)**
	**R**	**D**	**Q**	**R**	**D**	**Q**	
*flp-11* vs N2	1.910	1.202	0.379	0.3197	0.0071	4.26E-03	16, 7
*flp-11OE* vs N2	0.469	0.362	2.463	0.4163	3.05E-05	3.44E-05	17, 5
*aptf-1* vs N2	1.098	1.151	0.575	0.8023	0.0415	4.41E-02	27, 16
*flp-11* vs N2	2.032	1.155	0.137	0.1383	4.43E-03	2.78E-03	6, 8
*flp-11; egl-4(lf)* vs N2	20.234	0.829	0.279	0.0343	0.2178	9.55E-06	17, 8
*flp-11; egl-4(lf)* vs *flp-11*	9.956	0.718	2.026	0.0803	0.0467	0.2905	17, 6
*flp-11OE* vs *egl-4(lf)*	0.182	0.666	7.327	0.0490	0.1011	1.10E-05	8, 7
*egl-4(lf); flp-11OE* vs *egl-4(lf)*	0.764	1.041	1.125	0.3592	0.4691	7.74E-01	17,7
*flp-11OE* vs *egl-4(lf); flp-11OE*	0.238	0.640	6.511	6.06E-07	0.0033	4.44E-04	8, 17
*flp-11; egl-4(gf)* vs *flp-11*	0.075	0.489	4.699	0.0636	2.56E-12	1.37E-13	18, 7
*egl-4(gf)* vs *flp-11; egl-4(gf)*	1.684	0.632	1.260	0.9143	0.0002	5.92E-05	6, 18
*egl-4(gf)* vs *flp-11*	0.126	0.309	5.919	0.0329	5.92E-05	4.57E-09	6, 7
*daf-7; flp-11OE* vs *daf-7*	0.541	0.679	6.090	0.0627	1.5E-07	2.14E-11	18, 7
*flp-11OE* vs *daf-7*	0.899	0.820	3.636	0.9061	0.1030	7.61E-04	7, 7
*flp-11OE* vs *daf-7; flp-11OE*	1.662	1.208	0.597	0.4690	0.0326	7.69E-03	7, 18

### FLP-11 in RIS Acts Downstream of DAF-7 in ASI

In *C*. *elegans*, out of a total of 302 neurons, the ASI neuron is the key neuron to relay the animal’s metabolic state and is therefore an important regulator of developmental progression, feeding behavior, and aging processes. Under adverse conditions such as starvation, *C*. *elegans* larvae enter a developmental diapause known as the dauer larva (Hu, 2007; Baugh and Hu, 2020). In the presence of food, ASI prevents the animal from becoming dauer and thus promotes reproductive growth (Bargmann and Horvitz, 1991). Calorie restriction extends life span in an ASI dependent manner (Bishop and Guarente, 2007). ASI is the sole source of TGFβ under normal growth conditions, and upregulation of TGFβ in ASI when the animal is sated is required for satiety (Gallagher et al., 2013b).

Based on the role of ASI neuron in satiety quiescence, next we examined whether and how FLP-11 interacts with DAF-7 and ASI by testing a double mutant of *daf-7* mutant and *flp-11OE*. Overexpression of FLP-11 rescues the defect of *daf-7* mutants in satiety quiescence suggesting FLP-11 acting downstream of DAF-7 and ASI (Figure 2G).

### An RNAi Screen of 28 *ins* Genes Identified *ins-1* as a Negative Regulator for Satiety Quiescence

Next, we investigated the roles of *ins* genes in satiety quiescence, performing the same strategy of feeding RNAi combined with the Hidden Markov Model based analysis (Figure 3A and Table 3). RNAi of five *ins* genes altered satiety quiescence (Figure 3B): *1, 7, 23, 29*, and *39*. We arbitrarily set 0.6 and 1.7 as cutoffs for reduction and enhancement, respectively. While knockdown of *ins-1*, *7*, *29*, and *39* enhances satiety quiescence (*ins-1* reached statistical significance), knockdown of *ins-23* reduces satiety quiescence. The transition rates of *ins-1* and *ins-7* are shown in Figure 3C. The distribution of the values of each state of 28 independent control RNAi groups and the average values of transition rates show that the variation for quiescence duration is bigger and the animals have shorter quiescence bout compared to the *flp* RNAi screen. Nonetheless, the trend is consistent: the most groups are within 20% of the mean (with a few outliers), and although the animals are less quiescent, they spent most of their time either dwelling or quiescent (Figure 3D). Because INS-1 antagonizes the DAF-2 insulin receptor (Pierce et al., 2001), and because DAF-2 function is necessary for satiety quiescence (You et al., 2008), an increase of satiety quiescence by *ins-1* RNAi suggests that it inhibits satiety quiescence by suppressing DAF-2 activity.

**TABLE 3:** Normalized values of each state of roaming, dwelling and quiescence, the sample size, and statistical significance of 28 *ins* genes.

**Genes**	**Normalized values**			***p-*value (by Student’s *t*-test)**			**Sample size (*N*)**
	**R**	**D**	**Q**	**R**	**D**	**Q**	**Control, RNAi**
*ins-1*	0.04	0.64	1.94	0.0190	0.0002	0.0001	12, 22
*ins-2*	3.48	0.96	1.01	0.3049	0.8059	0.9659	8, 18
*ins-3*	0.72	0.95	1.33	0.7739	0.5973	0.4187	11, 20
*ins-4*	2.76	1.04	0.96	0.5994	0.8181	0.8130	7, 16
*ins-5*	0.44	1.04	1.30	0.2831	0.2501	0.1889	8, 16
*ins-6*	0.78	1.21	0.84	0.6907	0.1137	0.3120	12, 23
*ins-7*	0.24	0.78	1.66	0.1052	0.0356	0.0122	12, 21
*ins-8*	0.23	1.12	0.87	0.2596	0.3350	0.5606	9, 18
*ins-9*	0.42	0.98	1.18	0.1895	0.8851	0.5599	9, 18
*ins-10*	0.95	0.99	1.02	0.9430	0.9480	0.9437	7, 16
*ins-11*	0.73	0.95	1.23	0.4223	0.6071	0.2463	12, 24
*ins-13*	1.02	0.82	1.43	0.9845	0.0633	0.0818	8, 17
*ins-14*	0.37	1.20	1.12	0.0228	0.1293	0.5815	12, 23
*ins-15*	1.79	1.00	0.81	0.2619	0.9811	0.3658	11, 23
*ins-17*	0.87	0.90	1.17	0.7484	0.4055	0.3585	12, 18
*ins-21*	0.28	1.04	0.99	0.0878	0.7520	0.9619	9, 16
*ins-22*	1.03	0.86	1.24	0.9726	0.1179	0.1253	8, 17
*ins-23*	42.21	1.31	0.59	0.1942	0.0588	0.0500	6, 16
*ins-24*	0.65	1.06	0.92	0.5582	0.6025	0.6198	8, 16
*ins-25*	0.45	1.03	1.00	0.2041	0.7914	0.9819	6, 16
*ins-27*	0.38	0.97	1.09	0.2199	0.7788	0.7296	6, 15
*ins-29*	1.29	0.77	1.75	0.7624	0.0439	0.0537	8, 14
*ins-32*	0.37	1.13	0.79	0.2197	0.2675	0.3206	8, 18
*ins-33*	4.07	0.99	0.99	0.3313	0.9286	0.9639	9, 14
*ins-34*	0.23	0.83	1.35	0.1314	0.1200	0.0972	9, 17
*ins-35*	1.10	1.37	0.67	0.9182	0.0738	0.0811	7, 17
*ins-36*	0.13	0.83	1.29	0.0348	0.1040	0.0864	9, 18
*ins-39*	1.06	0.67	1.72	0.9486	0.0862	0.1125	8, 16

## Discussion

Among 28 tested *flp* genes, we found 10 FLPs that potentially regulate satiety quiescence either positively or negatively when the gene was knocked down by RNAi. Among them, *flp-11*, known to regulate other types of sleep-like behavior in *C. elegans* plays the most significant role in satiety quiescence. The *flp-11* mutation reduces satiety quiescence, whereas over-expression enhances it. FLP-11 controls satiety quiescence by acting in the RIS neurons; a mutant that carries a mutation in a gene *aptf-1*, a transcription factor that functions in RIS, is also defective in satiety quiescence. Genetic analyses suggest that FLP-11 acts upstream of the cGMP signaling and downstream of the TGFβ pathway.

From the RNAi screen of 28 *ins* genes, we identified *ins-1* as an antagonist for DAF-2 in satiety quiescence. *ins-1* is expressed multiple neurons in the nervous system, intestine and vulval muscles (Li and Kim, 2008). It is, however, noted to mediate starvation-induced salt learning by being released from AIA. Considering that ASI is the major neuron for satiety quiescence and that AIA neurons are the main target of ASI, we suggest that INS-1 serves as a negative feedback signal to reset ASI activity when the animal feeds continuously in an abundance of food. INS-7, the 2^nd^ best candidate, is expressed in the intestine and plays a role in aging by propagating FOXO signaling to other tissues (Murphy et al., 2007). Its expression in neurons is induced by *Pseudomonas* virulence (Kawli and Tan, 2008). Its downregulation is necessary for intermittent fasting-induced longevity (Honjoh et al., 2009). These findings suggest that INS-7 might mediate stress signals. The overexpression study of individual *ins* genes (Zheng et al., 2018) suggests *ins-7* could act as either an agonist or antagonist depending on the phenotype. Our results suggest, *ins-7* seems to act as an antagonist, as do *ins-29* and *ins-39* for satiety quiescence. In contrast, *ins-23* has been suggested to be an antagonist (Matsunaga et al., 2018) or neutral (Zheng et al., 2018), but it may act as an agonist for satiety quiescence. INS-7 is expressed in URX neurons and antagonizes INS-6 released from ASI neurons by converging on RIA interneurons which play an important role in head movement and turning by synapsing on SMD or RMD head motor neurons (Gray et al., 2005). This suggests that INS-7 might regulate satiety quiescence via controlling ASI function negatively as INS-1 does.

Although satiety quiescence has not been thoroughly examined for its homeostatic properties to be defined as sleep (Raizen et al., 2008), it contains a few key components of behavioral state of sleep, such as a distinct period of inactivity and reduction of sensory perception (You et al., 2008). In addition, it is, to our knowledge, the only sleep-like behavioral state regulated mainly by metabolic state without being associated with development progresses or harsh stresses; satiety quiescence can be induced without prior starvation or stress. Therefore, our RNAi screens using this behavior as a readout would provide an insight how neuropeptides might convey the information of the metabolic state of the body to induce a sleep-like state.

**FIGURE 4:**
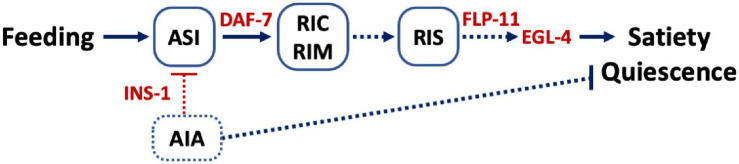
Model. Feeding activates ASI and promotes ASI to release DAF-7, the *C. elegans* TGFβ, which in turn activates the TGFβ receptors on RIM and RIC (Gallagher et al., 2013b). This activation leads to activation of RIS and release of FLP-11 to induce satiety quiescence. The cGMP signal initiated from ASI is also required for satiety quiescence. Although the exact sites of cGMP signal are unknown, our data suggest EGL-4 could act downstream of FLP-11. INS-1 presumably released from AIA could serve as a negative feedback signal to ASI and inhibit satiety quiescence. Lines indicate signaling that has been previously reported whereas dotted lines are hypothetical.

Based on our results, we propose a model (Figure 4) where neuropeptides regulate satiety quiescence. In *C. elegans*, out of a total of 302 neurons, the ASI neuron is the key neuron to relay the animal’s metabolic state and is therefore an important regulator of developmental progression, feeding behavior and aging processes (Bargmann and Horvitz, 1991; Bishop and Guarente, 2007; Hu, 2007; Baugh and Hu, 2020). ASI is the sole source of TGFβ DAF-7 under normal growth conditions. Upregulation of DAF-7 in ASI when the animal is sated is required for satiety quiescence. The receptor DAF-1 functions in RIM and RIC to promote satiety quiescence, connecting ASI to RIM and RIC (Gallagher et al., 2013b). RIC and RIM release octopamine and tyramine (Alkema et al., 2005) and therefore potentially regulate wakefulness. In fact, RIM synapses on RIS and activate or inhibit RIS depending on RIM’s activity level (Maluck et al., 2020). Optogenetic activation of RIS results in inhibition of locomotion and pharyngeal pumping, implicating that RIS is required for executing and maintaining sleep (Steuer Costa et al., 2019). Our study suggests that ASI provides another input to RIS through DAF-7 and DAF-1 acting on RIM and RIC and convey the animal’s nutritional status to promote a sleep-like state induced by satiety. Taken together, our study provides an insight into understanding how neuropeptides regulate sleep-like behavior by unveiling the conserved molecular mechanisms and the underlying neural circuit.

## Data Availability Statement

The raw data supporting the conclusions of this article will be made available by the authors, without undue reservation.

## Author Contributions

Y-JY conceived of the study and drafted the manuscript. MM and JK helped to design experiments. MM, EU, RS, JK, and Y-JY performed the experiments. All authors contributed to the article and approved the submitted version.

## Conflict of Interest

The authors declare that the research was conducted in the absence of any commercial or financial relationships that could be construed as a potential conflict of interest.
